# Pulmonary actinomycosis mimicking lung cancer on ^18^F-fluorodeoxyglucose positron emission tomography: a case report

**DOI:** 10.1186/s13256-022-03481-w

**Published:** 2022-07-01

**Authors:** Shinichi Miyazaki, Takeo Fujito, Yuki Kondo, Yasumasa Kuno, Shunsuke Mori, Ryo Yamashita, Junzo Ishida, Yoshiharu Nara, Takuya Ikeda

**Affiliations:** 1 Department of Respiratory Medicine, Yokkaichi Municipal Hospital, 2-2-37, Shibata, Yokkaichi-shi, Mie 510-0822 Japan; 2 Department of Orthopaedic Surgery, Yokkaichi Municipal Hospital, 2-2-37, Shibata, Yokkaichi-shi, Mie 510-0822 Japan; 3 Department of General Thoracic Surgery, Yokkaichi Municipal Hospital, 2-2-37, Shibata, Yokkaichi-shi, Mie 510-0822 Japan; 4 Department of Pathology, Yokkaichi Municipal Hospital, 2-2-37, Shibata, Yokkaichi-shi, Mie 510-0822 Japan

**Keywords:** Actinomycosis, Lung cancer, ^18^F-fluorodeoxyglucose positron emission tomography

## Abstract

**Background:**

Pulmonary actinomycosis is a chronic disease characterized by abscess formation, draining sinuses, fistulae, and tissue fibrosis. It can mimic other conditions, particularly malignant and granulomatous diseases, and is perhaps extremely challenging to diagnose.

**Case presentation:**

A 64-year-old Japanese man presented with 6-week history of a painful solid lump in the chest wall. Chest computed tomography scan revealed a mass-like consolidation in the left upper lobe, with rib erosion and direct extension into the anterior chest wall. ^18^F-fluorodeoxyglucose positron emission tomography scan showed increased metabolic activity in the mass, which is indicative of primary lung cancer. The bronchoscopy and computed tomography scan-guided transthoracic biopsy results were considered nondiagnostic. Finally, the patient was diagnosed with pulmonary actinomycosis via surgical resection. He completed an 8-week course of antibiotic therapy and experienced no recurrence.

**Conclusions:**

There is no difference in positron emission tomography/computed tomography scan findings between actinomycosis and malignancy. Therefore, pulmonary actinomycosis should be considered in the differential diagnosis of cases involving intensive activity on ^18^F-fluorodeoxyglucose positron emission tomography scan.

## Background

Actinomycosis is a rare, chronic granulomatous infection caused by *Actinomyces*, which are filamentous Gram-positive obligate anaerobic rod-shaped bacteria that normally colonize the mouth, colon, and vagina. Actinomycosis remains a diagnostic challenge because it is uncommon, physician experience with its clinical manifestations is limited, and culture of the causative microorganism is technically difficult. Pulmonary actinomycosis accounts for approximately 15% of all actinomycosis cases [1]. Aspiration of organisms from the oropharynx is the common cause of infection. Pulmonary actinomycosis is characterized by chest pain, fever, weight loss, hemoptysis, and cough [2]. Its typical radiographic feature is either the presence of a mass or pneumonia with or without pleural involvement [3]. Due to the nonspecific nature of its clinical presentation, the condition is often misdiagnosed as lung cancer. Therefore, diagnosis of pulmonary actinomycosis should be based on a combination of several factors, including positive culture results, presence of sulfur granules in the purulent matter from infected tissues, clinical and radiological features, and response to antibiotic treatment. The prognosis of this condition is excellent, and therapy with high-dose penicillin for 2–6 weeks, followed by amoxicillin for 6–12 months, is recommended.

We describe herein a patient with pulmonary actinomycosis mimicking lung cancer on ^18^F-fluorodeoxyglucose positron emission tomography (FDG-PET). Only a few case reports have documented pulmonary actinomycosis assessed via FDG-PET.

## Case presentation

A 64-year-old Japanese man presented to our department due to 6-week history of a painful solid lump in the chest wall. Moreover, he experienced loss of appetite and weight reduction. However, fever, night sweat, and respiratory symptoms were not observed. The patient was a former smoker (20 pack-years) but had no history of drinking. His medical history included hypertension, dyslipidemia, diabetes mellitus, and Basedow’s disease. Moreover, he was taking medications including telmisartan, amlodipine, atorvastatin, sitagliptin, metformin, and thiamazole.

**Fig. 1:**
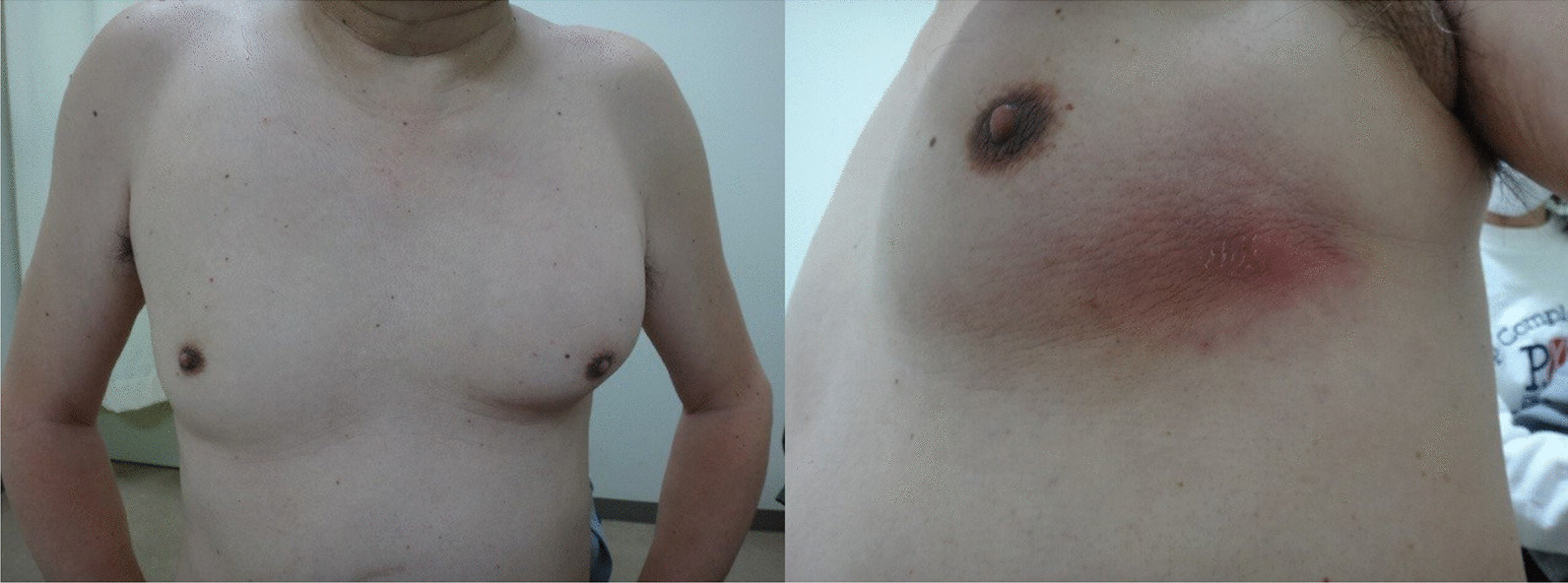
Physical examination findings of the chest wall

Physical examination revealed the following vital signs: temperature, 36.7 °C; blood pressure, 116/83 mmHg; heart rate, 109 beats/minute; respiratory rate, 20 cycles/minute; and oxygen saturation, 93% on room air. A large solid, nontender mass was palpated in the left anterior chest wall, and skin bruising was observed (Fig. 1). The patient had poor oral hygiene due to periodontitis. However, his other physical examination findings were normal.

**Fig. 2:**
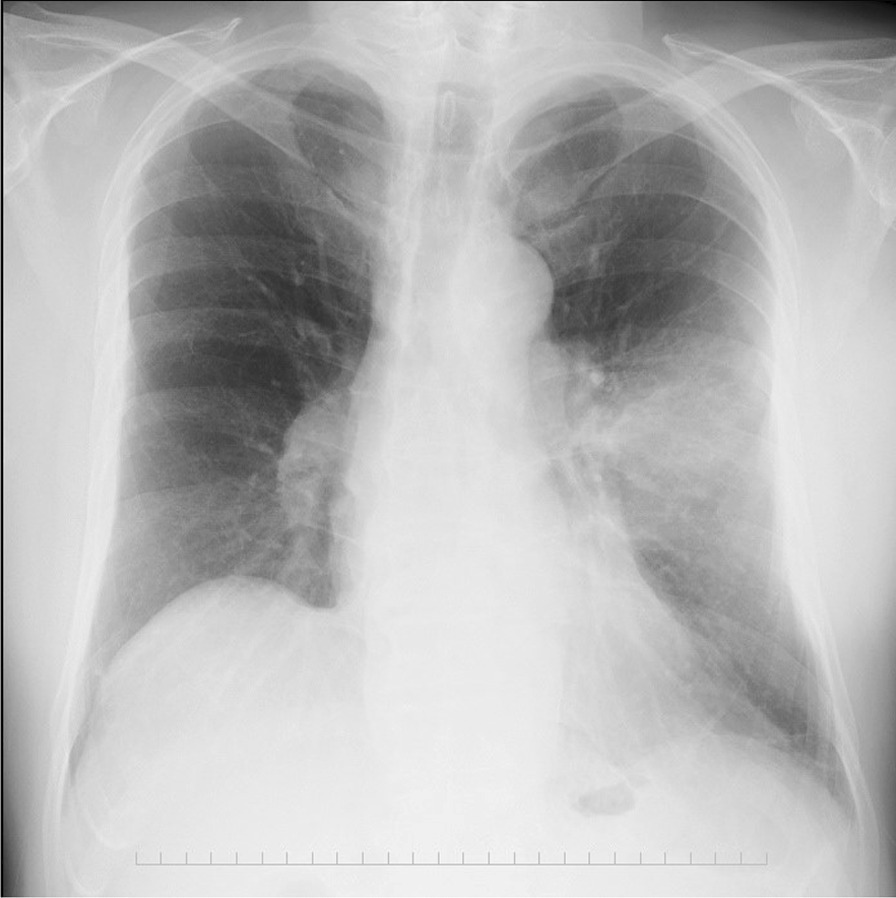
Chest radiography image upon presentation

**Fig. 3:**
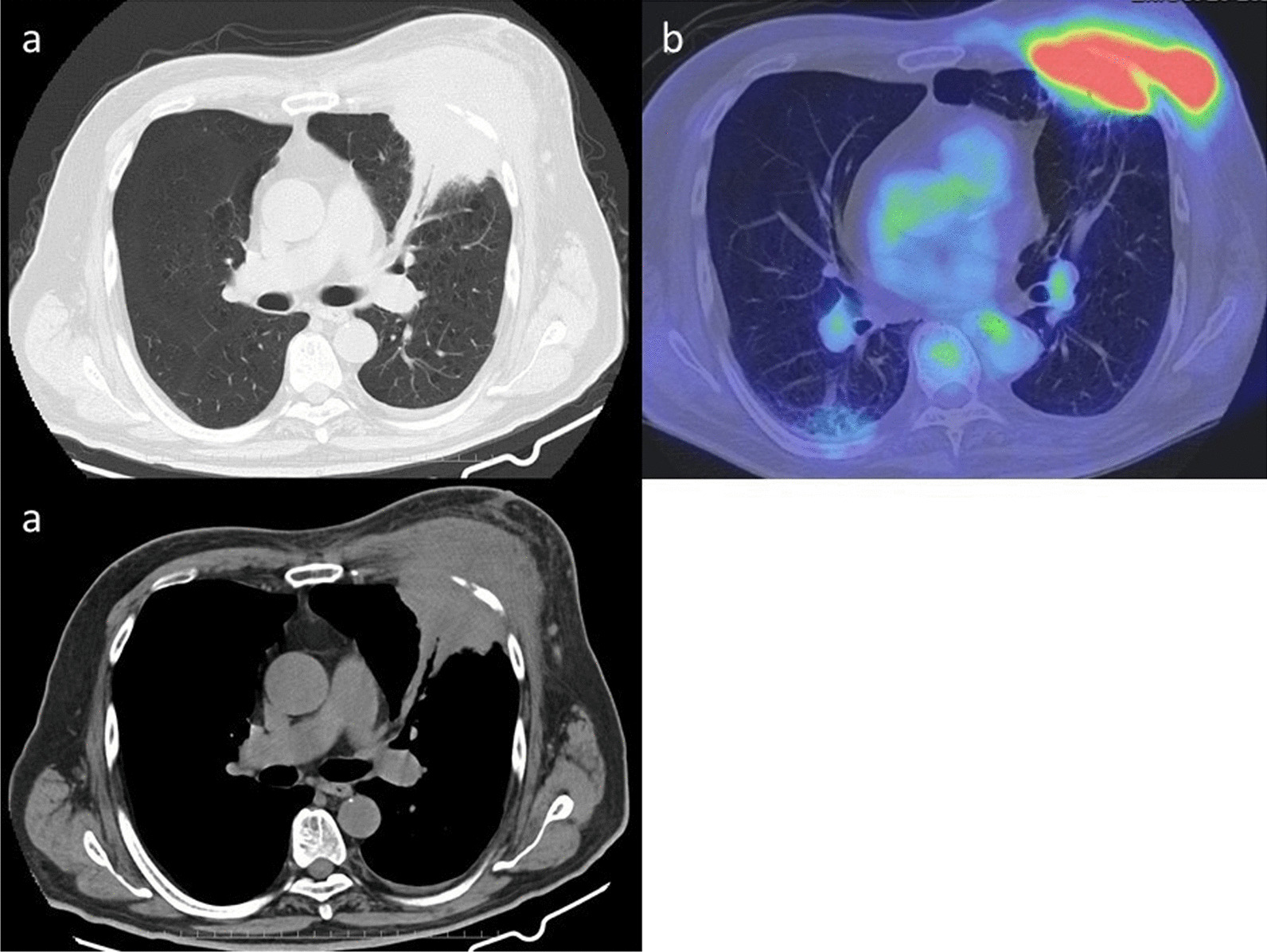
Computed tomography scan image of the chest (**a**) and ^18^F-fluorodeoxyglucose positron emission tomography image (**b**) upon presentation

The hemogram results were as follows: leukocytosis of 13.1 × 10^3^ cells/μL with left shift. The blood electrolyte levels and renal and liver function test results were normal. The C-reactive protein level was 178 mg/L. Hemoglobin A1c was 7.0%, and the thyroid function was normal. Blood cultures revealed no growth. Chest radiography revealed consolidation in the left middle lung (Fig. 2). Chest computed tomography (CT) scan showed a mass-like consolidation in the left upper lobe (Fig. 3a). Consolidation caused rib erosion, and it extended directly into the anterior chest wall, deep to the pectoralis major muscle. FDG-PET scan showed increased metabolic activity (maximal standardized uptake value [SUV] of 24.6) in the mass (Fig. 3b). A primary lung tumor invading the chest wall was suspected. The patient underwent bronchoscopy with bronchial washing and transbronchial biopsy, which did not reveal endobronchial abnormalities. After the procedure, the patient developed fever, and prophylactic antibiotic (amoxicillin/clavulanate 500 mg/125 mg orally every 8 h for 5 days) was administered. Biopsy using a specimen collected from the lesion in the left upper lobe had unremarkable results. Culture of the bronchial washing sample was sterile. CT scan-guided transthoracic biopsy of the mass was performed. Histologic examination revealed granulomatous tissues. Because the transthoracic biopsy result was considered nondiagnostic, surgical resection was conducted for diagnostic purposes.

**Fig. 4:**
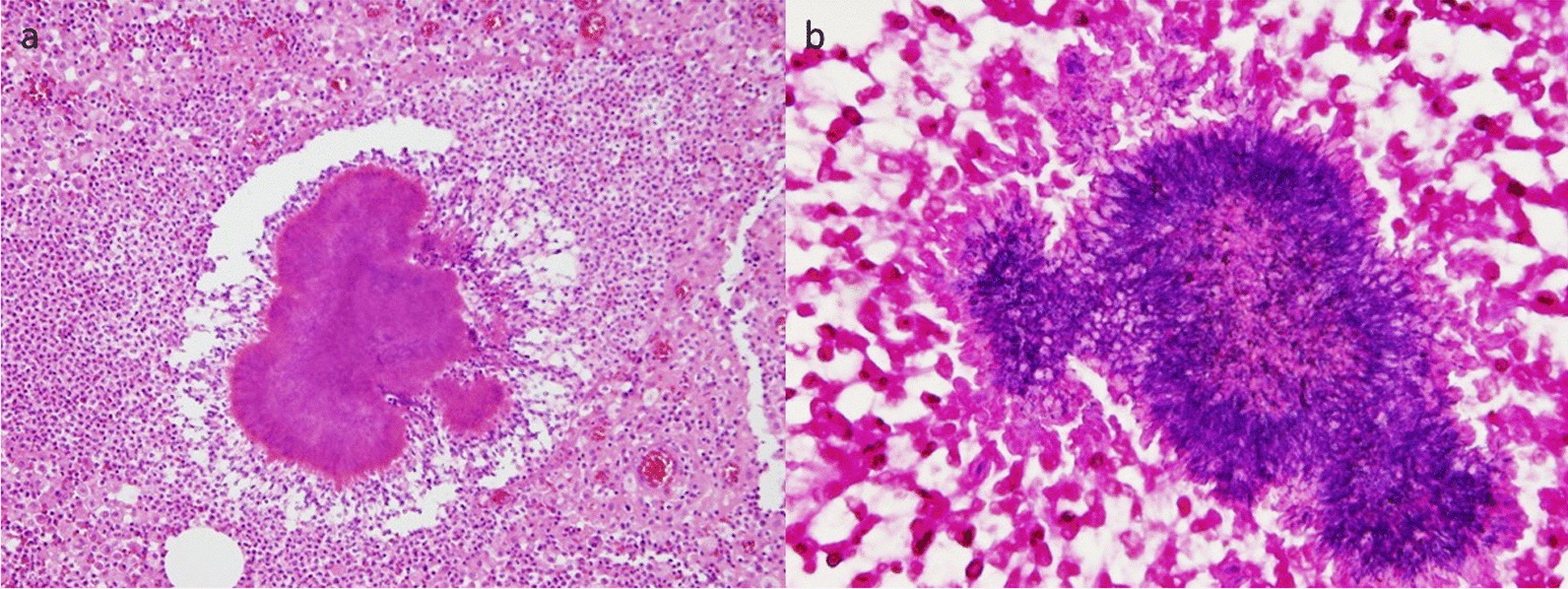
Results of the histopathological examination conducted using a surgical specimen. **a** Hematoxylin and eosin staining (original magnification, ×100). **b** Gram staining (original magnification, ×400)

Surgical extirpation of the mass with left upper lobe partial resection was performed. Pathological examination revealed the presence of sulfur granules and filamentous, Gram-positive rod-shaped bacteria in the inflammatory site (Fig. 4). Tissue cultures for bacteria, fungus, and mycobacteria were negative. After the diagnosis of actinomycosis was made, the patient was treated with amoxicillin (1000 mg orally every 8 h). He completed an 8-week course of antibiotic treatment and experienced no recurrence 2.5 years after the operation.

## Discussion and conclusions

We report herein a patient with pulmonary actinomycosis mimicking lung cancer on FDG-PET. To obtain a definitive diagnosis of pulmonary actinomycetes, isolation, identification, and culture of microorganisms are required. In this case, the culture results were negative because of the anaerobic nature of *Actinomyces*, prior antibiotic therapy, and use of inadequate culture techniques. In case of post-bronchoscopy fever [4, 5], use of prophylactic antibiotics is not recommended according to the guidelines of the British Thoracic Society [6]. Moreover, tracheobronchial stenosis was considered an independent risk factor for post-bronchoscopy pneumonia via a multivariate analysis in a case–control study [7]. Inappropriate use of antibiotics may contribute to false-negative culture results.

The incidence of actinomycosis has significantly decreased within the last three to four decades, and the clinical features are less aggressive compared with those in the preantibiotic era [8]. Proper dental hygiene and early antimicrobial treatment of infections are contributing factors. However, recently, only a few studies have assessed the clinical characteristics of pulmonary actinomycosis. In a case series of 94 Asian patients with pulmonary actinomycosis during the first decade of the twenty-first century [9], the median age was 57.7 (range 31–83) years, and the male:female ratio was 66:28. In total, 50 patients had history of smoking, and 45 patients presented with underlying pulmonary comorbidities such as infections caused by mycobacteria (*n* = 21), bronchiectasis (*n* = 18), and chronic obstructive pulmonary disease (*n* = 10). The nonpulmonary comorbidities were diabetes mellitus (*n* = 18), hypertension (*n* = 18), and alcohol abuse (*n* = 16). The presenting symptoms were cough (77.7%), hemoptysis (64.9%), and sputum secretion (61.7%). The chest CT scan findings were consolidation (74.5%), mediastinal or hilar lymph node enlargement (29.8%), atelectasis (28.7%), cavitation (23.4%), ground-glass opacity (14.9%), and pleural effusion (9.6%). Based on the clinical and radiological findings, lung cancer (35.1%) was the initial diagnosis, followed by pneumonia (19.1%) and mycobacterium infection (17.0%). The confirmatory diagnosis was obtained via surgical biopsy (*n* = 47), bronchoscopic biopsy (*n* = 24), and percutaneous transthoracic needle biopsy/aspiration (*n* = 23). Intravenous, followed by oral, antibiotic treatment was initiated after the diagnosis of actinomycosis. The mean durations of treatment with intravenous and oral antibiotics were 14.7 (range: 1–56) days and 153.2 (range: 5–672) days, respectively. Intravenous antibiotics, mostly comprising penicillin G (34.1%), cephalosporin (27.3%), ampicillin/sulbactam (7.9%), and amoxicillin (5.7%), were administered. The indications for surgical resection (*n* = 49) were persistent hemoptysis (*n* = 22), differential diagnosis of pulmonary malignancy (*n* = 17), and absence of radiologic response despite medical treatment (*n* = 4). In total, 92 patients completely recovered, and only 2 died from complications.

Actinomycosis is a rare condition, and physicians have limited experience in assessing its clinical manifestations. Moreover, laboratory cultivation and identification are challenging. Therefore, this condition is still difficult to diagnose. With consideration of the risk of actinomycosis, this infection can be diagnosed using less invasive methods, and unnecessary surgeries can be prevented. Only a few case reports have documented pulmonary actinomycosis assessed via FDG-PET [10, 11]. In 11 patients with pathologically confirmed pulmonary actinomycosis, the median maximal SUV on PET-CT scan increased to 5.5 (interquartile range: 4.2–8.8). However, this was higher than the threshold value of 2.5 that indicates malignancy [12]. The diagnostic value of FDG-PET in ruling out malignancy is limited.

We report herein the case of a patient with pulmonary actinomycosis mimicking lung cancer. This infection is rarely considered by pathologists when establishing a diagnosis. Because there is no difference in PET/CT findings between actinomycosis and lung cancer, the diagnostic value of FDG-PET is limited. If patients have fever after bronchoscopy, careful observation is generally recommended, and inappropriate use of antibiotics may lead to false-negative culture results.

## Data Availability

Data are available from the corresponding author on reasonable request.

## Abbreviations

FDG-PET: ^18^F-fluorodeoxyglucose positron emission tomography
SUV: Standardized uptake value
CT: Computed tomography
